# Antibacterial and Sterilizing Effect of Benzylpenicillin in Tuberculosis

**DOI:** 10.1128/AAC.02232-17

**Published:** 2018-01-25

**Authors:** Devyani Deshpande, Shashikant Srivastava, Paula Bendet, Katherine R. Martin, Kayle N. Cirrincione, Pooi S. Lee, Jotam G. Pasipanodya, Keertan Dheda, Tawanda Gumbo

**Affiliations:** a Center for Infectious Diseases Research and Experimental Therapeutics, Baylor Research Institute, Baylor University Medical Center, Dallas, Texas, USA; b Lung Infection and Immunity Unit, Division of Pulmonology and UCT Lung Institute, Department of Medicine, University of Cape Town, Cape Town, South Africa

**Keywords:** avibactam, benzylpenicillin, childhood tuberculosis, pharmacodynamics, pregnancy, tuberculosis

## Abstract

The modern chemotherapy era started with Fleming's discovery of benzylpenicillin. He demonstrated that benzylpenicillin did not kill Mycobacterium tuberculosis. In this study, we found that >64 mg/liter of static benzylpenicillin concentrations killed 1.16 to 1.43 log_10_ CFU/ml below starting inoculum of extracellular and intracellular M. tuberculosis over 7 days. When we added the β-lactamase inhibitor avibactam, benzylpenicillin maximal kill (*E*_max_) of extracellular log-phase-growth M. tuberculosis was 6.80 ± 0.45 log_10_ CFU/ml at a 50% effective concentration (EC_50_) of 15.11 ± 2.31 mg/liter, while for intracellular M. tuberculosis it was 2.42 ± 0.14 log_10_ CFU/ml at an EC_50_ of 6.70 ± 0.56 mg/liter. The median penicillin (plus avibactam) MIC against South African clinical M. tuberculosis strains (80% either multidrug or extensively drug resistant) was 2 mg/liter. We mimicked human-like benzylpenicillin and avibactam concentration-time profiles in the hollow-fiber model of tuberculosis (HFS-TB). The percent time above the MIC was linked to effect, with an optimal exposure of ≥65%. At optimal exposure in the HFS-TB, the bactericidal activity in log-phase-growth M. tuberculosis was 1.44 log_10_ CFU/ml/day, while 3.28 log_10_ CFU/ml of intracellular M. tuberculosis was killed over 3 weeks. In an 8-week HFS-TB study of nonreplicating persistent M. tuberculosis, penicillin-avibactam alone and the drug combination of isoniazid, rifampin, and pyrazinamide both killed >7.0 log_10_ CFU/ml. Monte Carlo simulations of 10,000 preterm infants with disseminated disease identified an optimal dose of 10,000 U/kg (of body weight)/h, while for pregnant women or nonpregnant adults with pulmonary tuberculosis the optimal dose was 25,000 U/kg/h, by continuous intravenous infusion. Penicillin-avibactam should be examined for effect in pregnant women and infants with drug-resistant tuberculosis, to replace injectable ototoxic and teratogenic second-line drugs.

## INTRODUCTION

The discovery of benzylpenicillin by Alexander Fleming in 1928 was an important primer of the modern medical era (1). Given that tuberculosis (TB) was the major problem of the time, the drug was tried on cultures of Mycobacterium tuberculosis by Fleming and others, but it was found to be ineffective by most (2–4). Penicillin kills bacteria by inhibiting d-alanyl–d-alanine-cleaving peptidase (d,d-transpeptidase), which catalyzes formation of the d,d 4→3 cross-linkage in peptidoglycan during cell wall synthesis. However, the predominant l,d 3→3 cross-linkage in M. tuberculosis, catalyzed by l,d-transpeptidases Ldt_Mt1_ and Ldt_Mt2_, is resistant to penicillin (5–7). A second reason for poor penicillin effect could be M. tuberculosis's class A β-lactamase, BlaC (8, 9). Semisynthetic penicillins, such as amoxicillin, as well as other β-lactam classes, such as carbapenems and cephalosporins, have been examined in combination with the β-lactamase inhibitor clavulanate, which confers an effect against M. tuberculosis (10–14). However, the inhibition of M. tuberculosis BlaC by clavulanate is slow and inefficient. We recently demonstrated that the non-β-lactam BlaC inhibitor avibactam endowed ceftazidime, which otherwise had no antimycobacterial effect, with dramatic efficacy against M. tuberculosis and Mycobacterium avium complex (15–17). Moreover, the targets inhibited by cephalosporins, carbapenems, and semisysnthetic penicillins in M. tuberculosis should not be assumed to be the same as for benzylpenicillins. Finally, the original benzylpenicillins themselves have not been examined in combination with β-lactamase inhibitors. In this study, we confined the definition of penicillins to either a benzylpenicillin (procaine or benzathine) or phenoxymethylpenicillin (penicillin V), *sensu strictu*. We hypothesized that avibactam could also confer susceptibility of M. tuberculosis to penicillin, if a lack of efficacy was due solely to β-lactamases and not lack of a penicillin-binding target.

M. tuberculosis organisms in TB lung cavities exist as a mix of different metabolic profiles: logarithmic-growth-phase bacteria, intracellular bacilli, semidormant bacilli under acidic conditions (SDB), and nonreplicating persisters (NRP) (18). NRP overexpress Ldt_Mt2_; thus, if the Ldt_Mt2_ hypothesis for the lack of penicillin effect is true, then NRP and SDB will be even more resistant to penicillin (6, 19). Indeed, because of these subpopulations of log-phase bacteria, SDB, and NRP, and because current first line anti-TB drugs are differentially effective against each one of these subpopulations, TB therapy relies on drug combinations. Microbial kill of log-phase M. tuberculosis defines bactericidal effect, and the average rate of kill in first 2 days is termed early bactericidal activity (EBA); the highest EBA is from isoniazid, at 0.6 log_10_ CFU per ml per day (20, 21). Microbial kill of either SDB by pyrazinamide or NRP by rifampin is considered a sterilizing effect and occurs at 0.15 log_10_ CFU/ml/day for rifampin and 0.10 log_10_ CFU/ml/day for pyrazinamide (18, 20, 22, 23). We have found that the same bactericidal and sterilizing effect kill rates and patterns are reflected in our hollow-fiber model of TB (HFS-TB) (20, 21, 23–26). Efficacy and optimal drug exposures of antibiotics derived in the HFS-TB have a forecasting accuracy of up to 94% in humans for bactericidal effect, EBA, and sterilizing effect, suggesting that if a drug is efficacious in the model, it likely has good clinical effectiveness (25, 27). Indeed, the HFS-TB is a European Medicines Agency (EMA)-approved and FDA-endorsed drug evaluation and selection methodology (28–30). In this study, we examined the bactericidal and sterilizing effect potential of penicillin plus avibactam in an *in vitro* culture system and the HFS-TB and found the combination to be highly effective against all four different M. tuberculosis subpopulations.

## RESULTS

### Benzylpenicillin plus avibactam is highly bactericidal in 12-well plates.

**FIG 1:**
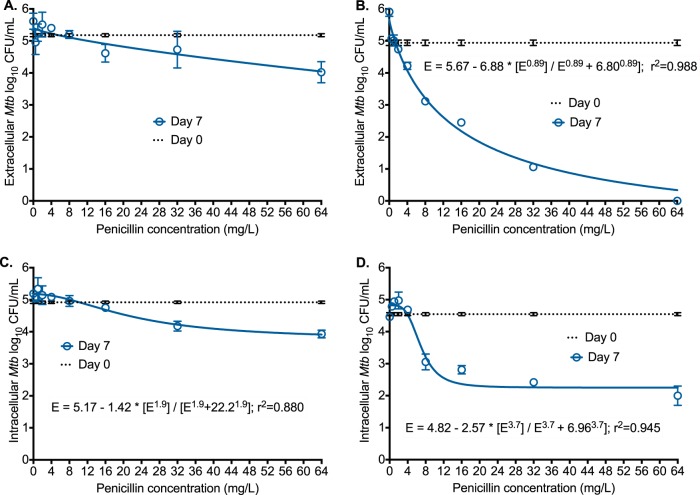
Effect of penicillin with and without avibactam at static concentrations. Error bars indicate SDs for three replicates. Cultures and drugs were coincubated for 7 days at 37°C under 5% CO_2_ and nonshaking conditions. The relationship between penicillin concentration (*C*) and Mycobacterium tuberculosis (*Mtb*) burden as log_10_ CFU/milliliter was analyzed for microbial effect (*E*) in log_10_ CFU/milliliter using the inhibitory sigmoid *E*_max_ equation, which takes the form *E* = *E*_con_ − *E*_max_ × *C*^H^/(*C*^H^ + *C*_50_^H^), where *E*_con_ is M. tuberculosis burden in nontreated controls, *E*_max_ is maximal kill, EC_50_ is potency or concentration mediating 50% of *E*_max_, and H is the Hill slope. (A) When penicillin alone was coincubated with extracellular M. tuberculosis, there was no kill at low concentrations, until around 64 mg/liter. There was poor convergence of the curve (*r*^2^ = 0.61), with an EC_50_ of 16,027 mg/liter. (B) Addition of avibactam dramatically increased maximum kill and improved potency >2,300-fold, to 6.8 mg/liter. (C) Penicillin killed intracellular M. tuberculosis at concentrations of ≥32 mg/liter, with an EC_50_ of 22.2 mg/liter. (D) Addition of avibactam improved the potency to 6.96 mg/liter and the maximal kill greater than 10-fold.

On the suggestion from K. Dheda to minimize bias and improve confidence of findings, all investigators and laboratory personnel (except D. Deshpande and T. Gumbo) were blinded to the identity of penicillin in all our experiments. Penicillin was designated drug X until the final result was reported. We coincubated log-phase-growth M. tuberculosis H37Ra with 7 different concentrations of penicillin alone in Middlebrook 7H9 broth with 10% oleic acid, albumin, dextrose, and catalase (OADC), with results shown in Fig. 1A. Penicillin did not kill M. tuberculosis until around 64 mg/liter, when it killed the bacteria 1.16 log_10_ CFU/ml below the level present on day 0. An absence of effect at low concentrations but some effect at higher concentrations suggests that penicillin's effect is inhibited by a saturable process that can be overcome at high concentrations. Next, we repeated the same experiment but this time examined all penicillin concentrations in the presence of 15 mg/liter of avibactam, leading to results shown in Fig. 1B. Penicillin maximal kill was 6.80 ± 0.45 log_10_ CFU/ml, with a 50% effective concentration (EC_50_) of 15.11 ± 2.31 mg/liter (*r*^2^ = 0.988). This maximal kill equals that of rifampin but exceeds that of either isoniazid or pyrazinamide in the same assay (17). The EC_50_ is easily achieved with standard benzylpenicillin dosing: injection or short infusion of 5 million international units (MIU) achieves a peak penicillin concentration of 400 mg/liter in serum (600 mg = 1 MIU). Next, we performed the same experiment but this time with adherent THP-1 monocytes infected with M. tuberculosis H37Ra in 12-well plates. Maximal microbial kill by the penicillin alone was 1.43 ± 0.29 log_10_ CFU/ml (Fig. 1C), with an EC_50_ of 22.26 ± 6.22 mg/liter. With addition of avibactam, the maximal kill increased to 2.42 ± 0.14 log_10_ CFU/ml and the EC_50_ decreased to 6.70 ± 0.56 mg/liter, which means that avibactam improved both efficacy (maximal kill) and potency (EC_50_). Thus, β-lactamase inhibition alone leads to dramatic microbial kill by penicillin.

### Benzylpenicillin is efficacious against intracellular M. tuberculosis in the HFS-TB and generates little resistance.

**FIG 2:**
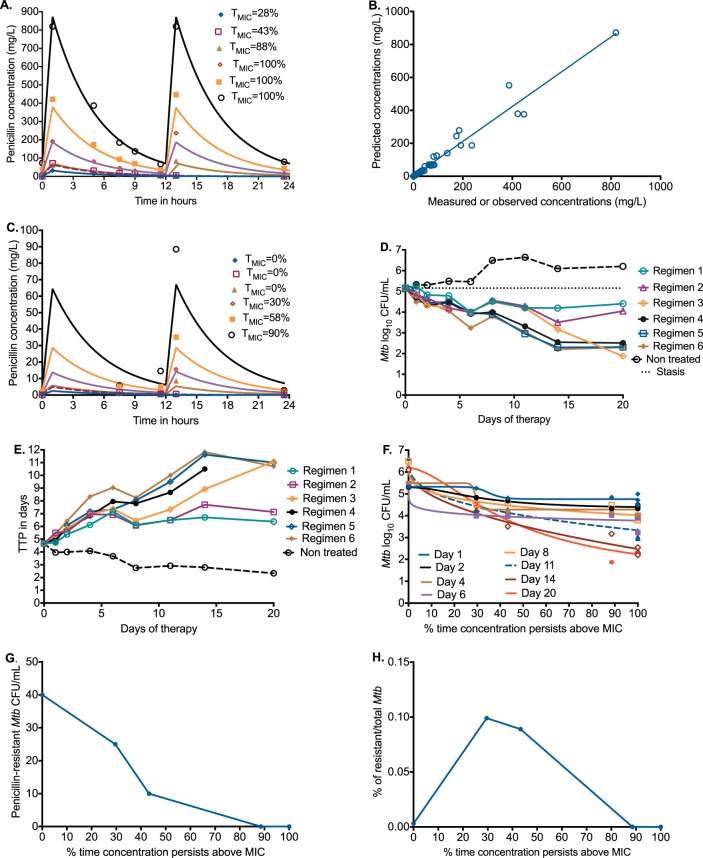
Penicillin dose scheduling study for intracellular M. tuberculosis. (A) Extracellular concentration-time profiles (symbols) shown against intended curves, demonstrating that intended pharmacokinetics were achieved. Doses were administered as a 1-h infusion. Regimen 1 achieved an extracellular *T*_MIC_ of 28% from a human-equivalent dose of 0.41 MIU administered once a day, regimen 2 a *T*_MIC_ of 43% from a human-equivalent dose of 0.9 MIU administered once a day, and regimen 3 a *T*_MIC_ of 88% from a human-equivalent dose of 0.8 MIU administered every 12 h; regimens 4 to 6 achieved a *T*_MIC_ of 100% from human-equivalent doses of 2.4 MIU administered every 12 h (regimen 4), 5.3 MIU administered every 12 h (regimen 5), and 10.2 MIU administered every 12 h (regimen 6). (B) The observed versus model predicted slope was 1.06 ± 0.03 (*r*^2^ = 0.972), indicating minimal bias. (C) Measured intracellular concentrations within infected monocytes were much lower than extracellular concentrations. Regimens 1 to 3 have an intracellular *T*_MIC_ of 0%, regimen 4 has a *T*_MIC_ of 30%, regimen 5 has a *T*_MIC_ of 58%, and regimen 6 has a *T*_MIC_ of 90%. (D) Time-kill curves demonstrate that all penicillin doses (except nontreated) killed below the day 0 burden (stasis), with no rebound growth. (E) A similar pattern was observed using TTP. (F) The relationship between bacterial burden and extracellular *T*_MIC_ is shown for each sampling day as M. tuberculosis in nontreated HFS-TB replicates continued to grow. Maximal kill (*E*_max_) is the span between nontreated controls and the maximal-effect portion of each curve. (G) The size of the drug-resistant subpopulation was highest in nontreated controls, which decreased with increasing *T*_MIC_. (H) However, when measured as a percentage of the total population, the drug-resistant subpopulation percentage versus exposure was best described as an inverted “U” curve with amplification of the penicillin-resistant subpopulation at a *T*_MIC_ of 30 to 40%.

Next, we performed dose-response studies against M. tuberculosis H37Ra-infected THP-1 human-derived monocytes in the HFS-TB. In the presence of avibactam, the penicillin MIC for M. tuberculosis H37Ra was 8 mg/liter. We wanted to establish the effect of benzylpenicillin-avibactam against intracellular M. tuberculosis, as well as to identify the best dosing schedule and optimal penicillin exposure at the same time. Thus, we examined three penicillin dosing schedules: once-a-day doses infused over 1 h, twice-a-day doses infused over 1 h every 12 h, and two continuous infusion doses over 24 h. We measured the concentration-time profiles in each HFS-TB, with results shown in Fig. 2A. The penicillin concentrations shown in Fig. 2A are well within those achieved in patients treated either with a 5-MIU injection or a 24-MIU continuous infusion over 24 h. Pharmacokinetic modeling of the measured concentrations revealed an elimination rate constant (mean ± standard deviation) of 0.227 ± 0.007 per hour (Fig. 2B). We also measured the concentrations of penicillin inside the M. tuberculosis-infected monocytes, which revealed the intracellular concentration-time profiles shown in Fig. 2C. The intracellular penicillin elimination rate constant of 0.213 ± 0.008 per hour differed slightly from the extracellular elimination rate, which means that the lower intracellular concentrations were likely driven by poor penetration into the human cells and not increased intracellular elimination. Avibactam extracellular concentrations were constant throughout at 13.54 ± 0.26 mg/liter, per design (slope = 0; *P* = 0.297).

Figure 2D and E show that all penicillin regimens killed the intracellular M. tuberculosis to a level below the day 0 bacterial burden. Maximal kill was 3.28 log_10_ CFU/ml in 20 days (Fig. 2D). We also simultaneously examined bacterial burden based on time to positivity (TTP) using the mycobacterial growth indicator tube assay (MGIT), a more sensitive assay than CFU. Figure 2E shows that the largest increase in TTP was 2.54-fold higher than on day 0, in the same range as with the three-drug combination first-line therapy in the past (31). Table 1 shows that microbial kill was highest when the proportion of time that concentration persisted above the MIC (*T*_MIC_) was highest, and that this was unrelated to peak concentration. Figure 2F shows that the *T*_MIC_ associated with maximal kill was ≥65% of 24 h (*r*^2^ = 0.967). This means that when penicillin doses are administered, the aim should be to exceed ≥65% of the day with concentrations above the MIC for maximal effect in TB.

**TABLE 1:** *r*^2^ for exposure index versus Mycobacterium tuberculosis burden^*a*^

Sampling day	Peak/MIC	AUC/MIC	*T*_MIC_ (%)
1	0.602	0.262	**0.741**
2	0.682	0.936	**0.966**
4	0.698	0.876	**0.9**
6	**0.93**	0.905	0.85
8	**0.978**	0.915	0.95
11	0.968	0.882	**0.978**
14	**0.996**	0.905	**0.996**
20	0.863	0.782	**0.968**

a The highest *r*^2^ values were with *T*_MIC_ throughout most of the experiment. The numbers in bold indicate the PK/PD parameter with the highest *r*^2^ value for that sampling day.

We also captured the M. tuberculosis subpopulation resistant to 3 times the MIC in each HFS-TB unit, with results shown in Fig. 2G. Figure 2H shows the penicillin-resistant subpopulation as percentage of the total bacterial burden versus *T*_MIC_, which was a typical inverted “U” shaped curve shape that we have identified for first-line and second-line anti-TB drugs in the past (23, 24, 32, 33). Thus, the penicillin exposure-versus-resistance relationship is the same shape as for standard anti-TB drugs. A *T*_MIC_ of 100% completely suppressed the penicillin-resistant subpopulation (*r*^2^ = 0.975). However, since the penicillin-resistant subpopulation was below 1%, considered clinically significant, penicillin monotherapy was much less prone to resistance emergence than each of the four first-line drugs.

### Penicillin has a dramatic bactericidal effect in the hollow-fiber model.

**FIG 3:**
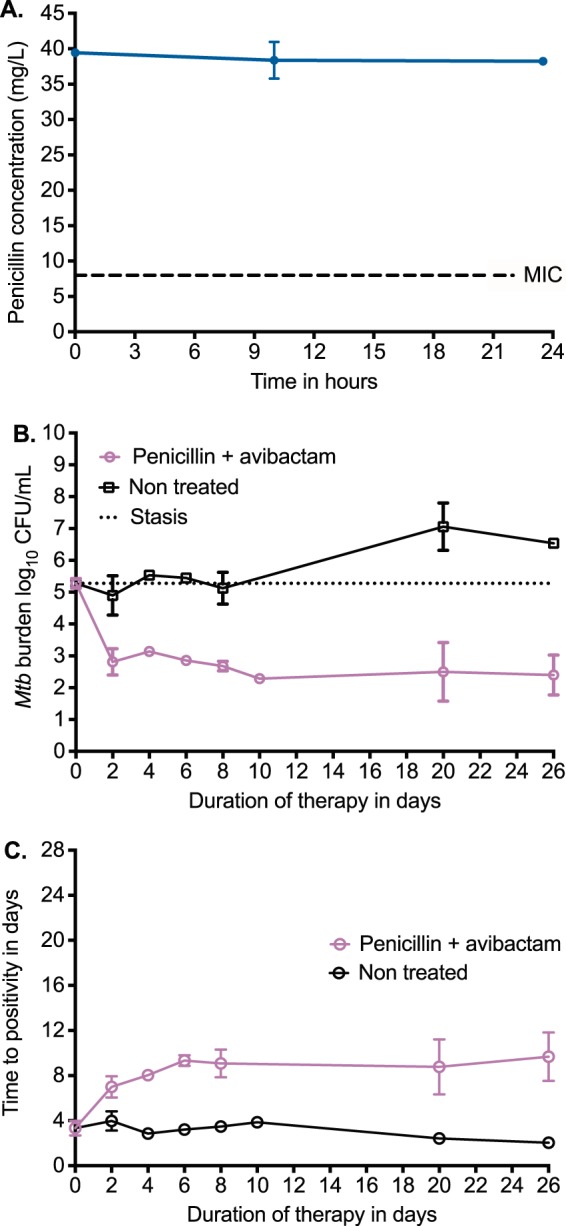
Bactericidal effect of continuous penicillin infusion. (A) As intended by this dosing strategy, the concentrations were kept at 5 times MIC for 100% of the 24-h dosing interval, which means the optimal dosing schedule for both microbial kill and resistance suppression. (B) Based on CFU counts, there was a rapid decline in bacterial burden the first 2 days, but instead of the usual rebound, the bacterial burden stayed constant for 4 weeks. (C) The same pattern was apparent when TTP was used to measure bacterial burden.

Next, we examined the efficacy of penicillin in the HFS-TB with log-phase-growth M. tuberculosis H37Ra using continuous infusion of penicillin equivalent to 1 MIU/h over 24 h (plus avibactam) to achieve 100% *T*_MIC_ over 4 weeks. Figure 3A shows the concentration-time profile achieved in duplicate HFS-TB, easily achieved in patients: as an example, the same 24 MIU of penicillin administered by continuous infusion over 24 h achieved a steady concentration of 80 mg/liter in serum. Figure 3B shows a surprising M. tuberculosis response curve with this regimen. First, there was a 2.88-log_10_ CFU/ml decline in only the first 2 days of therapy, or an EBA of 1.44 log_10_ CFU/ml/day. This EBA is 2.4 times that of isoniazid, the first-line drug with the highest EBA (24). Figure 3C demonstrates similar findings based on TTP, which is inversely proportional to bacterial burden. There was a 2.6-fold change in TTP on penicillin monotherapy in the HFS-TB. Second, Fig. 3B and C demonstrate a lack of rebound growth on monotherapy, not seen until now with any first-line drugs (24, 32, 34). This confirms that at a *T*_MIC_ of 100%, there was suppression of both penicillin-resistant and penicillin-tolerant M. tuberculosis over 4 weeks of therapy.

### Penicillin has a sterilizing effect against nonreplicating bacilli.

**FIG 4:**
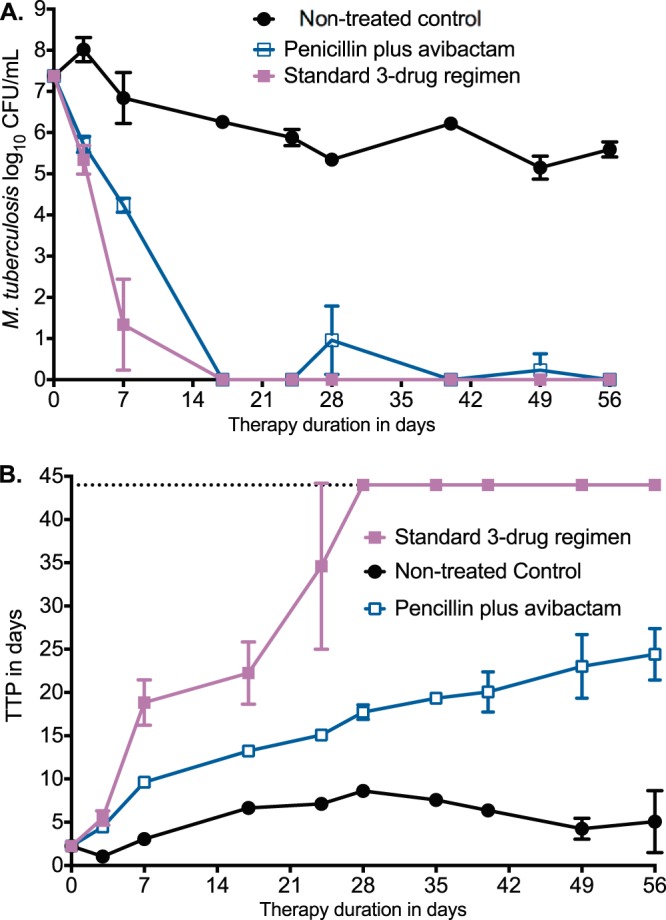
Sterilizing effect of penicillin-avibactam versus standard therapy All error bars indicate SDs for replicate HFS-TB units. (A) The decline in CFU/milliliter in the nontreated controls was because of the volumes removed from the systems and number of times which we sampled the peripheral compartment over 8 weeks of the experiment, which reduces the bacterial burden in a bacterial population that is not replicating or growing. Based on the changes in CFU/milliliter changes, the effect of penicillin plus avibactam was similar to that of the standard 3-drug regimen. (B) Based on TTP, the standard regimen reached culture negativity on day 28, which was faster than penicillin-avibactam.

The traditional dogma is that penicillin microbial kill stops in stationary-phase bacteria and is best with rapidly multiplying bacteria; thus, penicillin should not be effective against NRP or SDB (35). Moreover, penicillin is labile at low pH, and some extracellular SDB in lung cavities are at low pH (36–38). We compared the effect of penicillin (at a *T*_MIC_ of 100%) plus avibactam versus the standard three-drug regimen of isoniazid, rifampin, and pyrazinamide in the HFS-TB NRP model based on the streptomycin-dependent auxotroph M. tuberculosis SS18b in acidified (pH = 5.8) Middlebrook broth (23, 39). The M. tuberculosis strain penicillin MIC was 16 mg/liter, the rifampin MIC was 0.0625 mg/liter, the isoniazid MIC was 0.0156 mg/liter, and the pyrazinamide MIC was 12.5 mg/liter. M. tuberculosis was inoculated into triplicate HFS-TB after >10 days of streptomycin deprivation; the HFS-TB broth lacked streptomycin throughout the experiment. We measured the antibiotic concentrations achieved in each HFS-TB, which revealed exposures shown in Table 2. Figure 4A show the dramatic effect of penicillin-avibactam as a single agent, which achieved kill below limits of detection (in CFU per milliliter) at the same rate as combination standard therapy. Thus, penicillin killed >7 log_10_ CFU/ml of nonreplicating bacteria at acidic pH in the presence of avibactam, which was higher than the concentration for log-phase-growth bacteria. However, when we used the more sensitive TTP readout shown in Fig. 4B, the combination therapy reached negative cultures faster than penicillin-avibactam.

**TABLE 2:** Exposures of anti-TB drugs achieved in HFS-TB replicates

Antibiotic	MIC (mg/liter)	*T*_MIC_ (%)	AUC/MIC ratio	Peak/MIC ratio
Penicillin	16.0	100	587.6	27.52
Rifampin	0.06	100	915.40	86.66
Isoniazid	0.25	100	305.20	27.90
Pyrazinamide	12.5	100	110.00	7.09

### Penicillin is additive and antagonistic to ceftazidime-avibactam.

**FIG 5:**
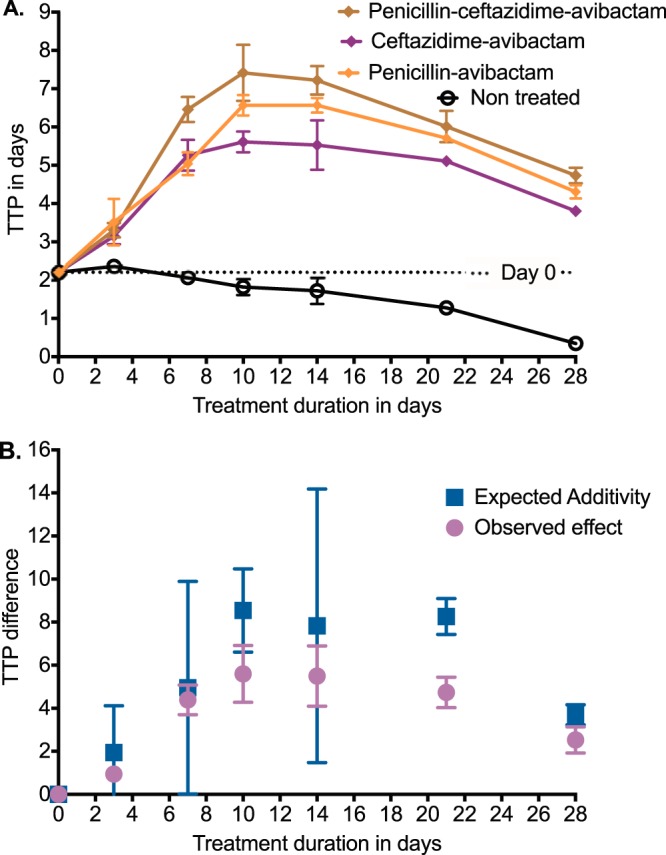
Additivity and antagonism of ceftazidime-avibactam with penicillin. (A) Error bars indicate SDs; where error bars are not visible, they were smaller than the symbols used. Data are expressed as TTP, which is a more sensitive assay than CFU. The nontreated controls grew well, as shown by decreasing TTP as duration of therapy increased. The combination of penicillin-ceftazidime-avibactam had the highest TTP on each sampling day, indicating the lowest bacterial burden. (B) Error bars are 95% confidence intervals. The bacterial burden on day of sampling minus that on day 0 gives the TTP change or effect. The effect observed with the combination was compared to that for ceftazidime-avibactam and penicillin-avibactam added together (expected additivity), which revealed overlapping 95% confidence intervals during the first 2 weeks. On day 21, the observed effect was less than expected additivity, as shown by nonoverlapping 95% confidence intervals for the triplicate HFS-TB. This means antagonism. However, the effect narrowed again on day 28.

Avibactam is currently coformulated with ceftazidime; thus, ceftazidime-avibactam is the most immediate source of avibactam for clinical use. However, ceftazidime-avibactam itself has very good anti-TB activity (17). We performed a HFS-TB with intracellular M. tuberculosis H37Ra to determine if the combination kills more than each agent alone. Figure 5A shows the bacterial burden as TTP over the 28 days of treatment. The nontreated controls had progressively declining TTP with time, indicating growth, while the combination of ceftazidime-avibactam plus penicillin had the highest TTPs, indicating the lowest bacterial burden. Effect or kill was defined as TTP on each sampling day minus that on day 0 (pretreatment TTP). The TTP effect of ceftazidime-avibactam alone on each day was added to that of penicillin-avibactam alone on that day to calculate the expected additivity, which was compared to the TTP effect in HFS-TB replicates treated with ceftazidime-avibactam plus penicillin (observed effect), based on Bliss independence definitions (40). Figure 5B shows that expected additivity was similar to observed effect in the first 14 days, since 95% confidence intervals overlapped. However, Fig. 5B shows that antagonism was encountered after day 21, since the observed combination effect TTP difference was higher than the expected additivity TTP difference. Surprisingly, the pattern changed again on day 28, so that expected additivity and observed effect were virtually similar. This means that additivity versus antagonism versus synergy definitions depended on when the sampling was done and that these change with duration of therapy. Nevertheless, Fig. 5B shows that the combination of ceftazidime-avibactam plus penicillin resulted in higher kill than did either drug alone.

### MDR-TB and incurable M. tuberculosis clinical strains are susceptible to penicillin.

**FIG 6:**
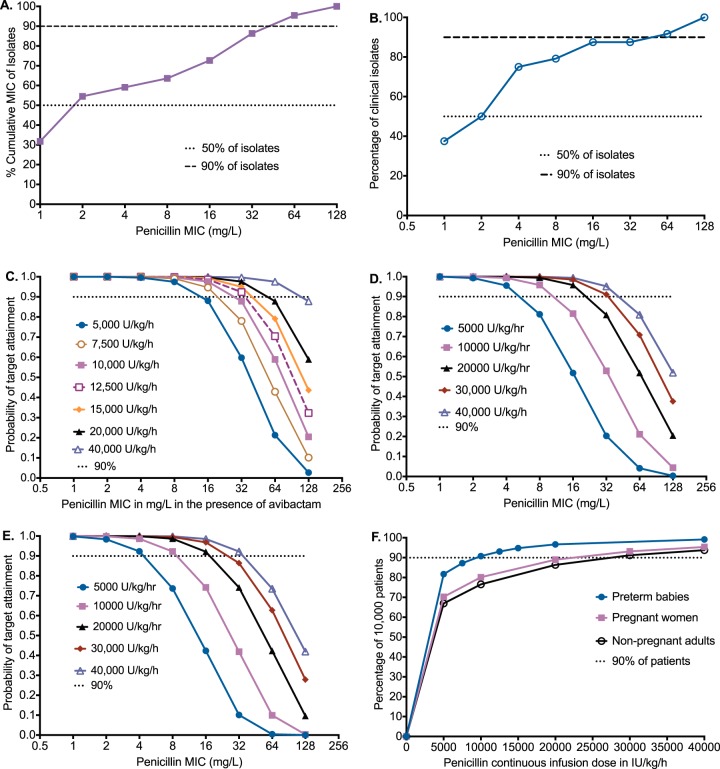
Monte Carlo experiments and MIC distributions for dose finding. (A) Cumulative MICs using reference method for MIC identification in clinical isolates that are predominantly from M/XDR-TB. Ninety percent of isolates had MICs below 64 mg/liter. (B) Cumulative MIC distribution based on MGIT assay. (C) Preterm children have relatively lower clearances and volumes of distribution, so higher concentrations are achieved and for longer than in adults at comparatively lower doses. Doses of 10 to 15,000 U/kg/h are relatively bunched up in performance, with a fall in PTA at an MIC of 64 mg/liter below 90% of patients. (D) In pregnant women in the third trimester, the PTA falls below 90% at MIC of 32 mg/liter with 20,000 U/kg/h and at an MIC of 64 mg/liter at 30,000 U/kg/h. (E) In adult patients not presently pregnant, the performance of different doses was similar to that in pregnant women. (F) The lowest doses above which 90% of patients achieve or exceed the 65% *T*_MIC_ target can be read from the graph and are 10,000U/kg/h for preterm children and 25,000 U/kg/h in adults. These are the doses that maximize bactericidal and sterilizing activity over the entire MIC distribution.

It could be that the penicillin effect is confined to M. tuberculosis laboratory strains and that penicillin would have no effect on clinical multidrug-resistant M. tuberculosis (MDR-TB) strains. Therefore, we sought to identify MICs for 25 clinical strains, a mixture of 20% drug-susceptible strains and 80% MDR-TB/extremely drug-resistant M. tuberculosis (XDR-TB) strains from South African patients. We examined this using two assays, the reference broth microdilution test and the MGIT. The penicillin MICs of the clinical strains are shown in Fig. 6A and B. The median MIC by either assay was 2 mg/liter, and the MIC_90_ was 64 mg/liter. Since penicillin concentrations higher than the MIC_90_ can be achieved in patients, these results mean that penicillin plus avibactam could be effective against a large proportion of MDR-TB and XDR-TB strains.

### The standard dose of penicillin would be effective in treating TB in the clinic.

Given this MIC distribution, how well would different doses of penicillin do in adult patients with pulmonary TB, in pregnant women in the third trimester, or in preterm neonates with disseminated TB in the clinic? Since TB clinical outcomes are mostly driven by pharmacokinetic and MIC variability (41–49), we utilized the penicillin population pharmacokinetic parameter estimates and interindividual variability identified by others (shown in Table 3) in 10,000-subject Monte Carlo experiments (MCE) in each of five doses (50–52). Our aim was to identify the dose of penicillin that would achieve the exposure of time above the MIC >65% in preterm babies with disseminated TB, pregnant women with pulmonary TB, and nonpregnant adults with pulmonary TB, treated using continuous infusion. Table 3 shows that the simulated population pharmacokinetic parameters for 5 doses each (total of 50,000 simulated subjects for each patient group) were similar to those in the initial domain of input, which is an internal validation step.

**TABLE 3:** Pharmacokinetic parameter estimates in domain of input versus 50,000-patient simulation output for each patient group

Group	Value for parameter in domain of input (% IIV)		Output in ≥50,000-subject simulation (% IIV)	
	Total clearance (liters/h/kg)	Central vol (liters/kg)	Total clearance (liters/h/kg)	Central vol (liters/kg)
Preterm infants^*a*^	0.10 (16)	0.36 (39)	0.10 (124)	0.36 (105)
Pregnant women	0.25 (28)	0.27 (70)	0.25 (106)	0.27 (159)
Nonpregnant adults	0.3 (40)	0.52 (104)	0.31 (45)	0.52 (111)

a In preterm infants, pharmacokinetic parameters were given for a two-compartment model, with domain of input versus mean value (interindividual variability [IIV]) of 0.774 liter/h/kg (40%) versus 0.733 liter/h/kg (102%) for intercompartmental clearance, and 0.152 liter/kg (17%) versus 0.151 liter/kg (105%) for peripheral volume.

Figure 6C shows the performance of each dosing regimen to achieve or exceed a *T*_MIC_>65% in infants of a gestational age of <32 weeks as the MIC increased. The probability of target attainment (PTA) remained above 90% until an MIC of 64 mg/liter in the highest dose in the children; even at an MIC of 128 mg/liter, the PTA for the highest dose fell slightly below 90%. Figure 6D shows results for pulmonary TB in pregnant women in the third trimester, which were similar to those in nonpregnant adults (Fig. 6E). Figure 6F is a summation which shows the proportion of patients who would achieve the *T*_MIC_ target of >65% when all MICs were taken into account. Based on Fig. 6F, the optimal dose in preterm children was 10,000 U/kg/h, while that in both pregnant and nonpregnant adults was 25,000 U/kg/h. Therefore, these are the doses to be administered in treatment of TB in these patient groups. In a sensitivity analysis, we determined the optimal doses in adults if penicillin penetration into lung lesions was only 50% (see Materials and Methods). The adult dose of 25,000 U/kg/h would still achieve or exceed the target exposure in >80% of adults. The performance of avibactam has been published before in our work on ceftazidime-avibactam (17).

## DISCUSSION

Benzylpenicillin and penicillin V are arguably the safest antibiotics used in the treatment of all infectious diseases. Billions of doses have been administered since the 1940s in diverse locales. The findings presented here suggest that penicillin-avibactam alone or penicillin in combination with ceftazidime-avibactam has the potential to improve both bactericidal and sterilizing effects and should be examined for the ability to shorten treatment duration as part of multidrug TB regimens in patients. Treatment shortening with reduction in relapse rates will impact transmission and disease burden and allow more efficient resource allocation; it is thus the research “holy grail” of TB treatment. The advent and spread of XDR-TB and incurable TB, and emerging treatment failures even with newer agents such as bedaquiline and delamanid, mean that newer and repurposed agents are urgently required to provide therapeutic options for these patients (53–55). MDR-TB and XDR-TB clinical isolates demonstrated good MICs; indeed, the true MICs may even be lower given that penicillin degrades at a rate of about 10% every 10 h in susceptibility test media, while M. tuberculosis has long doubling times and the MIC assay takes several days to complete, conditions that caused falsely higher MICs with another β-lactam, ertapenem (56–58). Because benzylpenicillin was never patented, and production costs have fallen over the decades, it costs very little and is readily available across the world. It represents one of the least expensive repurposed drugs for TB. On the other hand, continuous infusion imposes logistical and monetary costs on patients and TB programs, which could counterbalance the low costs. Moreover, avibactam may further increase the cost of this combination.

There has been recent community advocacy calling for stakeholders to design treatments for pregnant women with TB, given the dire need (59). TB in pregnant women is associated with a 2-fold increase in premature birth and low birth weight and a 6-fold-higher perinatal death rate (60). TB is responsible for 6% to 10% of all maternal mortality in low-HIV-prevalence settings and 15 to 34% in high-HIV-prevalence settings (60). There is currently no specific safe TB therapy targeted for this neglected and vulnerable population. Though the first-line drugs are considered to be fairly safe in pregnancy, there have been reports of teratogenicity from rifampin (61, 62). Even though current second-line drugs are known to adversely affect embryofetal development, the CDC still recommends them for treatment of pregnant women, after counseling the women, on the grounds that gains overwhelmingly outweigh the risks. Benzylpenicillin-avibactam could help cure the TB in these women, with reduced fetal toxicity risks. While penicillin crosses the placenta, it is not teratogenic, which has made it the “go to” drug for pregnant women with different infections. While patient studies are lacking for avibactam, the FDA label indicates that reproductive studies performed during pregnancy in animal showed no adverse effects on embryofetal development (63). Nevertheless, fetal toxicity from long-term administration of avibactam in people has not been adequately studied. Thus, penicillin-avibactam could represent a breakthrough for pregnant women suffering from TB.

Closely related is that congenital TB is an increasingly recognized problem, with the children often born preterm (64). In the case of MDR-TB in children <6 years old, the rates of ototoxicity with aminoglycosides are extremely high and devastating (65). Indeed, even in adults with MDR-TB, the ototoxicity rates are up to 70% with amikacin (66). Our finding that the use of the BlaC inhibitor avibactam allows penicillin to kill M. tuberculosis opens the door to immediately test penicillin-avibactam efficacy in children. Penicillin is also already the go-to drug in infectious diseases of neonates, infants, and toddlers. Our findings create opportunities to try combinations of other M. tuberculosis BlaC inhibitors with penicillin.

Next, we wanted to make recommendations for how benzylpenicillin could be used in neonates, pregnant women, and nonpregnant adults with TB, including in outpatient settings. First, penicillin degrades to <90% in 13 h after reconstitution in normal saline or sterile water for injection at room temperature, or when loaded onto a portable pump reservoir and infused at 37°C for 1 day (67). However, when benzylpenicillin was reconstituted with sodium citrate (a buffer) for the purpose of outpatient parenteral antimicrobial therapy, concentrations remained above 90% when the drug was stored at 3 to 5°C for 7 days and then kept at 37°C for 24 h (67, 75, 76). The outpatient strategy would be to use this buffer and keep the solution refrigerated at 3 to 5°C for 7 days, with the pump reservoir loaded daily. The recommend doses per day for a neonate, pregnant woman, and nonpregnant adults are shown in Table 4. If normal saline or water for injection is used as the diluent, the solution is refrigerated at 3 to 5°C for 7 days, and then the intravenous therapy would be loaded twice a day at doses shown in Table 4, taking into account the degradation rates, which could impose a hardship and likely necessitate inpatient care. Though the doses recommended are large, they are not far from those used for other severe infections. In children with congenital syphilis, for example, up to 300,000 U/kg/day (i.e., 12,500 U/kg/h) is used, and for adults the penicillin dose we recommend is only 1.5 to 1.75 times that for those with infective endocarditis.

**TABLE 4:** Recommended doses of benzylpenicillin for different patient populations

Patient group	Dose (U/kg/h) of benzylpenicillin	
	In sodium citrate buffer (continuous infusion), loaded once a day	In saline or sterile water, loaded morning and evening
Neonates	10,000	10,000
Pregnant women	25,000	25,000
Nonpregnant adults	25,000	25,000

Finally, the combination of ceftazidime-avibactam with penicillin resulted in higher kill rates than benzylpenicillin-avibactam alone. During early therapy the combination was additive, but later on it became antagonistic. This remarkable finding has implications on general pharmacology studies examining drugs for synergy and antagonism. Often, antibiotic pairs are presented as either antagonistic or synergistic. Here, we show that the same concentrations may be additive or antagonistic depending on duration of therapy! This is in addition to our prior findings in the HFS-TB and in patients that anti-TB agents could be antagonistic or synergistic at the same time point in a concentration-dependent fashion (42, 45, 46, 68). This means that definitions of synergy, additivity, and antagonism may need modification. The question of double β-lactam coverage for TB that we have proposed is open to debate. For Gram-negative bacilli, double β-lactam coverage is sometimes used on the theory that binding of each of the β-lactams in the combination is on unique penicillin-binding proteins. It remains to be seen whether this strategy will work for TB.

## MATERIALS AND METHODS

### Bacterial strains, cell lines, and growth conditions.

The following laboratory M. tuberculosis strains were used: H37Ra (ATCC 25177), H37Rv (ATCC 27294), and SS18b (donated by Stewart Cole), and 25 clinical strains from the South African Medical Research Council. Storage and culture conditions for log-phase bacteria, SDB, and intracellular M. tuberculosis were as described in our prior studies (23, 68, 69). M. tuberculosis SS18b was first cultured in Middlebrook 7H9 broth plus 10% OADC in the presence of 50 mg/liter of streptomycin to achieve log-phase growth, followed by subculture in the same medium but without streptomycin for 14 days. Human-derived THP-1 cells (ATCC TIB-202) grown in RPMI 1640 plus 10% fetal bovine serum FBS (RPMI-FBS) were infected with H37Ra for intracellular experiments in wells and HFS-TB using methods as described previously but with a coincubation period of 3 h.

### Materials and chemicals.

Penicillin was purchased from Baylor University Medical Center pharmacy (Dallas, TX). Avibactam was synthesized by BOC Sciences, Shirley, NY. Penicillin G-d7 was purchased from Toronto Research Chemicals (Ontario, Canada). Hollow-fiber cartridges were obtained from FiberCell (Frederick, MD).

### Identification of MICs.

MICs were identified on two different occasions each, using the standard broth macrodilution test and the MGIT. We used rifampin as a positive control for the drugs and M. tuberculosis H37Rv as the control for bacterial isolates. The final penicillin concentrations examined were 0, 0.5, 1, 2, 4, 8, 16, 32, 64, and 128 mg/liter, in combination with a final fixed avibactam concentration of 15 mg/liter in triplicate, which is the peak concentration achieved with a 0.5-g clinical dose of avibactam.

### HFS-TB model.

The construction of the HFS-TB has been described in detail previously (24, 31–34, 69–71). In all HFS-TB experiments, 20-ml M. tuberculosis cultures were inoculated into peripheral compartments of HFS-TB. Penicillin and avibactam were administered via computerized syringe pumps, based on dosing schedules described in legend of Figure 2. Sampling of the central compartment of each system for drug concentrations was performed at 0, 1, 5, 7.5, 9, 11.5, 13, and 23.5 h after the first dose or start of the penicillin infusion, for the intracellular M. tuberculosis studies. In log-phase and NRP experiments, the central compartment was sampled at 0, 10, and 23.5 h and 0, 1, 2, 3, 5, 11.5, 20, and 23.5 h after dose or start of infusion. Drug concentrations in the samples were measured as described below. The peripheral compartment was sampled at 0 (before first dose), 1, 2, 4, 6, 8, 11, 14, and 20 days of therapy for THP-1 cell counts, number of viable cells, THP-1 cell volume, and culturing of the intracellular M. tuberculosis, as described in the legend to Fig. 2. The peripheral compartment was sampled for bacterial cultures on days 0, 2, 4, 6, 8, 10, 14, 21, and 26 for the log-phase study and days 0, 3, 7, 17, 24, 28, 40, 49, and 56 for NRP study, and bacterial burden was identified using both CFU counts and TTP. Cultures of M. tuberculosis SS18b utilized Middlebrook 7H10 agar and broth supplemented with 50 mg/liter of streptomycin. Culture conditions were as described previously (24, 31–34, 69–71).

### Penicillin concentration assay.

Liquid chromatography-tandem mass spectrometry (LC-MS/MS) analysis was performed using a Waters Acquity ultraperformance liquid chromatograph (UPLC) coupled with Waters Xevo TQ mass spectrometer. Separation was achieved by injecting 2 μl of sample on a Waters Acquity UPLC HSS T3 column (50 × 2.1 mm; 1.8 μm) using a binary gradient. Solvents for UPLC were as follows: solvent A, 0.1% aqueous formic acid, and solvent B, 0.1% formic acid in acetonitrile. Samples were diluted 1:20 with internal standard solution containing penicillin G-d7. The transitions used were *m/z* 335 > 176 (penicillin G) and *m/z* 342 > 183 (penicillin G-d7). The between-day coefficient of variation (CV) for analysis of low and high (in parentheses) quality controls for RPMI-FBS were 2% (2%) and 2% (2%) for saline, and those for Middlebrook 7H9 broth were 4% (2%). The within-day CV for analysis of low and high (in parentheses) quality controls were 4% (2%) for RPMI-FBS, 2% (1%) for Middlebrook 7H9 broth, and 2% (2%) for saline. The lower limits of quantitation were 0.25 mg/liter for Middlebrook 7H9 broth for the nonreplicating persister and log-phase studies and 0.375 mg/liter in RPMI-FBS and 0.05 mg/liter for saline (to measure intracellular concentrations). The avibactam, rifampin, isoniazid, and pyrazinamide assays for concentration measurement have been described in detail previously (17, 71).

### Pharmacokinetic/pharmacodynamic modeling.

Compartmental pharmacokinetic modeling was employed for all HFS-TB studies, as described in detail previously (23, 24, 31, 33). Exposure response relationships were examined using the inhibitory sigmoid *E*_max_ model for microbial kill and the quadratic function for the relationship between drug-resistant subpopulation and exposure (23). All modeling was performed using ADAPT 5 software (Biomedical Simulations Resource, University of Southern California). Results were transferred to GraphPad Prism 7 for graphing purposes.

### Monte Carlo experiments.

Pharmacokinetic parameter estimates and interindividual variability as percent CV in preterm infants with a gestational age of ≤32 weeks were from Muller et al., those for pregnant women during the third trimester were from Johnson et al., and those for nonpregnant adults were from Komatsu et al., shown in Table 1 (50–52). In the case of disseminated TB in preterm neonates, the serum concentrations were used for the exposure calculations. Since the HFS-TB studies had been performed using media with serum and albumin, we made no further adjustments for protein binding. In the case of adult pulmonary TB, there are no recent proper pharmacokinetic studies on the epithelial lining fluid or infected lung tissue concentration of penicillin. In 1950, Jensen et al. (77) noted that the benzyl ester of penicillin achieved high concentrations in the lungs, unlike earlier penicillin. Similarly, studies by Ungar and Muggleton using mice and guinea pigs with bacterial pneumonia revealed that unlike normal lung, the concentration-time profiles achieved in infected lung tissue by benzylpenicillin exceeded those in serum >2-fold (72). Around the same time, Heathcote and Nassau noted that in patients treated with benzypenicillin who had lung resection, the lung tissue concentrations at the time of pulmonary artery ligation were 1- to 2-fold higher than in serum from simultaneous blood draw (73). We took a conservative approach and assumed a 1:1 ratio in effective concentration in lungs of TB patients versus serum, based on up to 2-fold drug penetration and 50% protein binding of penicillin. For sensitivity analysis, we assumed we were 50% overoptimistic in our assumptions based on ELF penetration calculated using compartmental pharmacokinetic approach for the ureidopenicillin, piperacillin (74). For each drug these population pharmacokinetic parameter estimates, and the covariance, were used as the domain of input in subroutine PRIOR of ADAPT. Simulations were run for each dose for 10,000 patients, for each group of patients, so that >50,000 patient pharmacokinetics were simulated for preterm children alone, pregnant women alone, and adults not presently pregnant (i.e., 160,000 in all). We generated 24-h concentration-time profiles for patients on continuous infusion with each dose, and then at each MIC we calculated the PTA for a *T*_MIC_ of>65%. In order to calculate the proportion of 10,000 patients who achieved this target when treated by each dose, we took an expectation over the entire MIC distribution, as detailed previously (17).
